# Characteristics of paramedian pontine arteries disease and its association with hemoglobinA1c

**DOI:** 10.1002/brb3.946

**Published:** 2018-02-28

**Authors:** Haiyan Li, Yaqing Shu, Biao Hu, Yongqiang Dai, Yinyao Lin, Yilong Shan, Yuge Wang, Wei Cai, Zhengqi Lu

**Affiliations:** ^1^ Department of Neurology The Third Affiliated Hospital of Sun Yat‐Sen University Guangzhou China; ^2^ Guangdong Provincial Bioengineering Institute (Guangzhou Sugarcane Industry Research Institute) Guangzhou China

**Keywords:** branch atheromatous disease, lipohyalinotic degenerative disease, paramedian pontine arteries, plasma hemoglobinA1c, prognosis

## Abstract

**Objectives:**

The association of branch atherosclerotic disease (BAD) and diabetes mellitus (DM) in the territory of posterior circulation is rarely discussed. Intracranial BAD was divided into two different types: paramedian pontine arteries (PPA) disease (PPD) and lenticulostriate arteries (LSA) disease. The goal of the study was to evaluate the clinical characteristics of PPD and its association with hemoglobinA1c (HbA1c) in China.

**Materials and Methods:**

Radiologically confirmed PPD was defined as an isolated unilateral infarction extending to the ventral surface of the pons. Small deep cerebral infarctions are usually caused by two different pathological changes of arteries: BAD and lipohyalinotic degeneration (LD). We compared the vascular risk factors between BAD and LD in PPA territory. A total of 159 stroke patients were analyzed (PPD,* n* = 75; LD,* n* = 84). Patients with PPD were also categorized into two groups according to follow‐up modified Rankin Scale (FmRS) scores. Logistic regression analyses were used for the evaluation of independent risk factors of PPD and prognosis.

**Results:**

Comparison between PPD and LD revealed statistical significance in fasting glucose, HbA1c, estimated glomerular filtration rate (eGFR), and uric acid (*p* = .011, *p* = .005, *p* = .027, *p* = .018, respectively). Compared with LD, PPD was only related to HbA1c (*p* = .011) in logistic regression analysis. There were statistically significant differences between the two groups based on the stratification of FmRS scores in fasting glucose, HbA1c, homocysteine, eGFR, and the occurrence of DM. After multivariate analysis, only HbA1c was related with poor prognosis of PPD (*p* = .002).

**Conclusions:**

The subtypes and prognosis of small deep brain infarcts are significantly influenced by elevated HbA1c level in PPA territory. DM might play an important role in the pathogenesis of PPD.

## 1 INTRODUCTION

Glycosylated hemoglobinA1c (HbA1c) reflects the average fasting and postprandial blood glucose over a period of 2 to 3 months and is clinically used to evaluate diabetes control (Egoldstein, 2004). During hospitalization, HbA1c was useful to identify undiagnosed diabetes mellitus (DM) and prediabetes, regardless of the specific blood glucose in acute ischemic stroke (Roquer et al., 2014).

Caplan suggested that intracranial branch atheromatous disease (BAD) was the fourth etiological factor of small deep cerebral infarction (Caplan, 1989). It is a common stroke subtype of Asian populations and has drawn more and more attention in recent years. BAD is caused by atherosclerotic plaques that make the orifice of larger caliber penetrating arteries narrow or occlusive (Fisher, 1979; Terai, Hori, Tamaki, & Saishoji, 2000). Intracranial BAD was divided into two different types: paramedian pontine arteries (PPA) disease (PPD) and lenticulostriate arteries (LSA) disease. Small deep cerebral infarctions are usually caused by two different pathological changes of arteries: BAD and lipohyalinotic degeneration (LD), which is mainly caused by lipid degeneration in the process of penetrating arteries (Baumgartner, Sidler, Mosso, & Georgiadis, 2003; Caplan, 1989).

Previous study showed that HbA1c was not correlated with the risk for various subtypes of ischemic stroke (Heo, Lee, Kim, Kang, & Yoon, 2010). Furthermore, our previous study, which represented all of acute ischemic stroke patients, showed that DM or HbA1c was not significantly related to progression and poor outcome of BAD (Men, Li, et al., 2013; Men, Wu, et al., 2013). However, Ichikawa et al. (2010, 2012) found DM had an independent association with the development of brainstem infarctions and higher HbA1c levels were independently correlated with the basilar artery/internal carotid artery ratio, which indicated that DM has different effects on anterior and posterior circulation (Ichikawa et al., 2009). Some studies concluded that BAD often results in fluctuations and progression of the disease (Bassetti, Bogousslavsky, & Barth, 1996; Kaps, Klostermann, Wessel, & Bruckmann, 1997). Although it was reported that various factors are associated with the progression of BAD (Yamamoto et al., 2011), the correlation between HbA1c and PPD or its prognosis was not discussed in those studies. We wondered whether HbA1c might influence only a type of BAD, such as PPD. To identify the traditional vascular risk factors that have different effects on PPD, we conducted a clinical and radiological investigation of the relationship of HbA1c levels with Chinese patients with PPD and evaluated whether HbA1c may be an independent risk factor for PPD vasculopathy and its prognosis.

## 2 METHODS

### 2.1 Patient selection

Between January 2008 and August 2015, 354 consecutive patients with a first‐ever acute brainstem infarctions, who had symptom onset within 7 days, were collected in the Department of Neurology of The Third Affiliated Hospital of Sun Yat‐sen University. We excluded patients who were seriously infected and whose stroke subtype was large artery disease (*n* = 100), cardio‐embolism (*n* = 16), and other determined or undetermined origin (*n* = 29). At admission, HbA1c was examined to exclude patients without laboratory data from HbA1c (*n* = 20) or lost during follow‐up (*n* = 30). Therefore, a total of 159 patients were eventually enrolled. The Hospital institutional review board approved this study (No. 2011‐2‐48). Patients or families signed informed consent. For research purposes, researchers record the patient's examination scores and standard laboratory test results.

### 2.2 Diagnosis of ischemic stroke subtype by MRI

**Figure 1:**
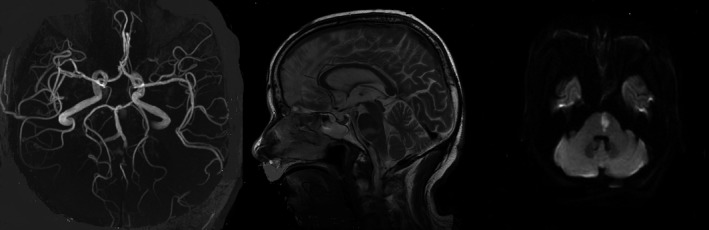
Images of paramedianpontine arteries disease

**Figure 2:**
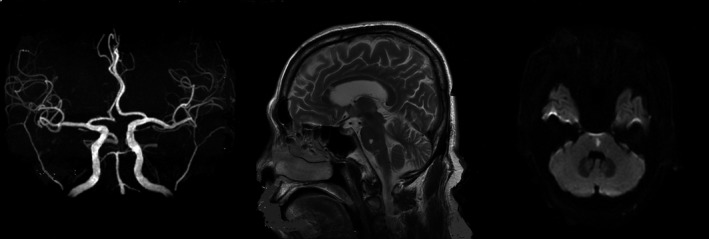
Images of lipohyalinotic degeneration(LD) in the pons

Small deep cerebral infarctions are usually caused by the two different pathological changes of arteries: 1. BAD and 2. LD. The diagnosis of BAD is based on radiological findings and previous reports (Yamamoto et al., 2011), defined as follows: the BAD of LSA is cerebral infarction with a diameter greater than 15 mm and three or more lesions are seen in axial slices, and PPA is defined as a unilateral infarction that extends to the basal surface of the pons (Figure 1). The PPA irrigates the medial and lateral parts, and the medial wall of the corticospinal tract, which includes anteromedial and anterolateral pontine arteries. We attempted to discriminate BAD, which is presumed to be caused atherosclerotic lesions of penetrating arteries of larger caliber at its orifices or proximal part, from LD (Figure 2), which is thought to result from lipohyalinosis of penetrating arteries (Baumgartner et al., 2003). We only selected BAD in PPA territory to study, which was known as PPD.

We classified other infarcts as LD in pontine arteries territory based on the clinicoanatomic correlations in patients with stroke (Men, Li, et al., 2013; Tatu, Moulin, Bogousslavsky, & Duvernoy, 1998). Tatu et al. (1998) used a map to determine the infarcts of the PPA territory based on arterial territory definition. Some patients deteriorate within 3 days of stroke onset and stroke radiological findings usually do not stabilize until approximately 3 days after onset. We performed two MRIs for each selected patient within 3 days and made the final diagnosis based on the second MRI.

### 2.3 Clinical information and assessment

Following risk factors or comorbid conditions were recorded: age, gender, hypertension, previous cerebrovascular accident (PCA), DM (defined as receiving diabetes medication, fasting glucose (FG) ≥126 mg/dl or 2 hr postprandial blood glucose≥200 mg/dl after achieving stable medical and neurological status) dyslipidemia, coronary artery disease (CAD), and smoking (>10 cigarettes/day). Hypertension was defined as follows: previously diagnosed, including blood pressure examinations from the last year, newly diagnosed (≥ 160/90 mmHg) at time of discharge or at least 1 week of hospital care, or current treatment during the last 2 weeks before stroke onset. Dyslipidemia was defined as follows: previously diagnosis, receiving cholesterol‐reducing agents, or newly diagnosis with fasting serum total cholesterol>240 mg/dl. CAD was defined as follows: patients with known angina or myocardial infarction. Clinical and laboratory data were collected from these adult individuals. To know kidney function, we used the estimated glomerular filtration rate (eGFR) equation to estimate glomerular filtration rate (GFR) by serum creatinine (S‐Cr) and age: eGFR (ml/min per 1.73 m^3^) = 194 ×  age ^− 0.287^ × S‐Cr ^− 1.094^ (if female × 0.739) (Ichikawa et al., 2009).

The National Institutes of Health Stroke Scale (NIHSS) scores at hospital admission were assessed immediately prior to MRI scans (Caplan, 1989). Next, we assessed the clinical outcome using a modified Rankin scale (mRS) score at 6 months (± 1 week) after the onset of the acute stroke (van Swieten, Koudstaal, Visser, Schouten, & van Gijn, 1988). We further divided patients with PPD into two groups with follow‐up mRS (FmRS) scores of >2 and ≤2. A mRS score of 6 was used for patients who died during the study period. Patients with FmRS ≤2 were considered to have a good outcome; patients with FmRS > 2 were considered to have a bad outcome (Bassetti et al., 1996) in PPD. Six months after stroke is the period for most functional recovery and is considered to be appropriate for assessing the outcome (Duncan, Jorgensen, & Wade, 2000). The factors described above were again compared between two groups.

MR images were obtained within 48 h of admission on a 1.5‐T whole‐body imager system (Signa; GE Medical Systems, Milwaukee, WI, USA). Intracranial vertebra‐basilar atherosclerotic diseases were assessed on MRA. Color‐coded Duplex sonography was used to detect intracranial carotid artery and vertebral artery stenosis.

### 2.4 Statistical analysis

Differences in continuous variables among groups were analyzed with Student *t* test or Kruskal–Wallis rank sum test. The χ^2^ test or Fisher exact test were used for categorical variables, as appropriate. Two‐tailed *p* values <.05 were subjected to logistic regression analyses. Multivariate analysis was used to adjust for determining risk factors and prognosis for PPD. The 95% confidence intervals (CI) were calculated, and *p* < .05 was considered statistically significant. Results were expressed as odds ratios (OR) and OR > 1.0 in bivariate analysis were chosen into multiple logistic regression analysis. The processing of data, including statistical calculations and preparing of tables, was made using the SPSS software version 16.

## 3 RESULTS

### 3.1 Comparison of clinical characteristics in PPD and LD

The baseline characteristics for the two groups (PPD and LD) are listed in Table 1. According to the second MRI, 75 patients were defined as PPD and 84 as LD. Patients enrolled included 97 men (61.0%) and 62 women (39.0%) with ages ranging from 40 to 95. The median score of NIHSS was 5 (interquartile range [IQR], 3–7) with an average at 5.36 ± 3.15. Among the 159 patients, 135 (84.9%) had hypertension, 74 (46.5%) had hyperlipidemia, 81 (50.9%) had DM, 30 (18.9%) were currently smoking, 46 (28.9%) had PCA, and 19 (11.9%) had CAD. 45 (28.3%) had basilar artery stenosis on MRA. In both groups, the initial NIHSS scores and poor outcomes at 6 months after acute stroke in the PPD group were significantly higher than those in the LD group (*p* < .05). Comparison between PPD and LD showed a statistically significant difference in FG, HbA1c, eGFR, and uric acid (*p* = .011, *p* = .005, *p* = .027, and *p* = .018, respectively; Table 1).

**Table 1:** Comparison of clinical characteristics in PPD and LD

Characteristics	All patients *N* = 159	PPD *N* = 75	LD *N* = 84	*p*
Age, (year)	65.91 ± 10.99	66.04 ± 11.60	65.79 ± 10.47	.885
Gender, male, no.(%)	97 (61.0)	44 (58.7)	53 (63.1)	.568
Risk factors, no.(%)				
Hypertension	135 (84.9)	63 (84.0)	72 (85.7)	.763
Diabetes mellitus	81 (50.9)	41 (54.7)	40 (47.6)	.375
Dyslipidemia	74 (46.5)	40 (53.3)	34 (40.5)	.105
Cigarette smoking	30 (18.9)	13 (17.3)	17 (20.2)	.640
Coronary artery disease	19 (11.9)	10 (13.3)	9 (10.7)	.611
Previous cerebrovascular accident	46 (28.9)	20 (26.7)	26 (31.0)	.552
Basilar artery stenosis	45 (28.3)	32 (42.7)	13 (15.5)	.000**
Initial NIHSS	5.36 ± 3.15	6.64 ± 3.55	4.21 ± 2.20	.000**
FmRS(>2 at 6 month after ictus)	36 (22.6)	27 (36.0)	9 (10.7)	.000**
Laboratory findings, mean ± SD				
Systolic pressure	159.59 ± 23.46	158.79 ± 22.53	160.31 ± 24.38	.684
Diastolic pressure	85.22 ± 13.29	83.73 ± 14.16	86.55 ± 12.39	.183
LDLC	3.41 ± 0.99	3.43 ± 0.95	3.39 ± 1.04	.632
HDLC	1.11 ± 0.26	1.11 ± 0.27	1.12 ± 0.25	.844
TC	5.13 ± 1.38	5.28 ± 1.68	4.99 ± 1.04	.258
TG	2.11 ± 1.69	2.25 ± 2.18	1.99 ± 1.07	.445
ApolipoproteinB	1.04 ± 0.30	1.08 ± 0.33	1.02 ± 0.26	.523
ApolipopretoinA‐1	1.32 ± 0.26	1.32 ± 0.26	1.33 ± 0.25	.909
FG	6.56 ± 2.23	7.02 ± 2.30	6.13 ± 2.10	.011*
HbA1C, (%)	6.86 ± 1.92	7.31 ± 2.11	6.46 ± 1.65	.005**
eGFR	0.51 ± 0.14	0.54 ± 0.16	0.49 ± 0.13	.027*
Uric acid	341.88 ± 99.60	322.05 ± 101.29	359.59 ± 95.20	.018*
Fibrinogen	3.75 ± 1.02	3.68 ± 1.24	4.28 ± 4.91	.464
Homocysteine	13.08 ± 4.37	12.50 ± 3.80	13.60 ± 4.80	.112

eGFR, estimated glomerular filtration rate; FG, fasting glucose; FmRS, follow‐up modified Rankin Scale scores; HbA1C, hemoglobinA1c; HDLC, high‐density lipoprotein cholesterol; LD, lipohyalinotic degeneration; LDLC, low‐density lipoprotein cholesterol; NIHSS, National Institutes of Health Stroke Scale; PPD, paramedian pontine arteries disease; TC, Total cholesterol; TG, triglyceride.

*p < .05; **p < .01.

### 3.2 Comparison of clinical information between two groups divided by FmRS scores in PPD

The differences in HbA1c, FG, Homocysteine, eGFR, and the occurrence of DM were statistically significant between the two groups established according to the stratification of FmRS scores. The patients with PPD in poor outcome group had increased frequency in DM (*p* = .011) and higher FG (*p* = .007), Homocysteine (*p* = .038), HbA1c (*p* = .016), and lower eGFR (*p* = .037) levels as compared to those in good outcome group (Table 2).

**Table 2:** Comparison of clinical information between two groups divided by FmRS scores in PPD

Variables	Good outcome group (*N* = 48) mRS ≤2	Poor outcome group (*N* = 27) mRS >2	*p*
Age, (year)	65.15 ± 10.58	67.63 ± 13.29	.377
Gender, male, no. (%)	29 (60.4)	15 (55.6)	.682
Risk factors, no. (%)			
Hypertension	41 (85.4)	22 (81.5)	.746
Diabetes Mellitus	21 (43.8)	20 (74.1)	.011*
Dyslipidemia	27 (56.2)	13 (48.1)	.500
Cigarette smoking	9 (18.8)	4 (14.8)	.759
Coronary artery disease	4 (8.3)	6 (22.2)	.154
Previous cerebrovascular accident	11 (22.9)	9 (33.3)	.327
Basilar artery stenosis	21 (43.8)	11 (40.7)	.800
Initial NIHSS	5.00 ± 2.12	9.56 ± 3.72	.000**
Laboratory findings, mean±*SD*			
Systolic pressure	156.98 ± 22.88	162.00 ± 21.95	.358
Diastolic pressure	82.85 ± 12.32	85.30 ± 17.09	.517
LDLC	3.42 ± 0.98	3.44 ± 0.91	.942
HDLC	1.10 ± 0.23	1.12 ± 0.34	.728
TC	5.01 ± 1.19	5.75 ± 2.27	.077
TG	2.13 ± 0.97	2.45 ± 3.43	.212
ApolipoproteinB	1.05 ± 0.28	1.12 ± 0.40	.732
ApolipoproteinA‐1	1.35 ± 0.27	1.25 ± 0.24	.105
FG	6.43 ± 1.82	8.09 ± 2.68	.007**
HbA1C, (%)	6.67 ± 1.19	8.45 ± 2.82	.011*
eGFR	0.51 ± 0.16	0.59 ± 0.15	.037*
Uric acid	339.91 ± 103.60	290.29 ± 90.35	.056
Fibrinogen	3.56 ± 0.80	4.03 ± 1.60	.210
Homocysteine	13.17 ± 3.73	11.29 ± 3.66	.038*

eGFR, estimated glomerular filtration rate; FG, fasting glucose; FmRS, follow‐up modified Rankin Scale scores; HbA1C, hemoglobinA1c; HDLC, high‐density lipoprotein cholesterol; LDLC, low‐density lipoprotein cholesterol; NIHSS, National Institutes of Health Stroke Scale; PPD, paramedian pontine arteries disease; TC, Total cholesterol; TG, triglyceride.

*p < .05; **p < .01.

### 3.3 Logistic regression model to identify the independent predictors for the risk factors and prognosis for PPD

Logistic regression analyses were employed to estimate the risk factors associated with PPD (Table 3), according to the differences significantly between PPD and LD, also those in prognosis of PPD after adjusting for confounders including eGFR, FG, Homocysteine, and HbA1c (HbA1c instead of prevalence of DM).

**Table 3:** Logistic regression analysis for the associated risk factors and prognosis of PPD

Parameters	Bivariate analyses		*p*	Multivariate analyses		*p*
	OR	95% CI for OR		OR	95% CI for OR	
Risk factors Of PPD						
FG	1.208	1.039–1.405	.014			
HbA1C, (%)	1.280	1.066–1.537	.008	1.256	1.043–1.513	.016
eGFR	13.134	1.268–136.075	.031	9.099	0.864–95.799	.066
Uric acid	0.996	0.993–0.999	.019			
Prognosis of PPD						
eGFR	28.837	1.050–791.806	.047	21.913	0.778–617.200	.070
HbA1C, (%)	1.561	1.184–2.058	.002	1.546	1.167–2.049	.002
FG	1.397	1.108–1.761	.005			
Homocysteine	0.869	0.758–0.996	.044			

CI,confidence interval; eGFR, estimated glomerular filtration rate; FG, fasting glucose; FmRS, follow‐up modified Rankin Scale scores; HbA1C, hemoglobinA1c; NIHSS, NIH stroke scale; OR, odd ratio; PPD, paramedian pontine arteries disease.

Only HbA1c (OR=1.256, *p* = .016) was an independent predictor for the occurrence of PPD. In addition, the HbA1c was independently associated with poor outcome Of PPD (OR=1.546, *p* = .002).

## 4 DISCUSSION

Risk factors and progressive motor deficits of all BAD have been widely researched. However, few studies have focused on prognosis of BAD in PPA territory and the risk factors of BAD in the territory, compared to LD. In this study, higher HbA1c level is strongly correlated with BAD in PPA territory, suggesting the potential involvement of HbA1c in the pathogenesis of BAD in PPA territory, compared to LD. It is to our knowledge that our study is the first time to show the correlation of Hb1Ac level change with the prognosis of BAD in PPA territory, compared to other risk factors.

HbA1c measurement is now widely used to monitor chronic hyperglycemia (Barr, Nathan, Meigs, & Singer, 2002). Moreover, HbA1c determination was used to identify new DM cases and could be useful not only for the diagnosis of DM in patients with acute hyperglycemia, but also for discovering DM in those with acute stroke at normal glucose levels (Roquer et al., 2014).

Some previous studies have shown that DM is associated with cerebral small vessel disease (CSVD) (Kloppenborg, Nederkoorn, Graaf, & Geerlings, 2011). A recent major study has shown that DM is correlated with specific patterns of stroke type, etiology, and topography and has a higher incidence of subcortical infarcts and more frequent effects on posterior circulation regions (Uluduz, Ince, & Bozluolcay, 2005). Previous neuropathological studies demonstrated that a high incidence of subcortical infarctions in patients with DM, especially in the pons perfused by PPA (Baumgartner et al., 2003; Terai et al, 2000). A recent study showed that branch occlusion was a more important mechanism in posterior circulation diseases (Kim et al., 2012). Based on these reports, this study indicated that PPD is independently associated with HbA1c, compared with LD, which represented the control of diabetes. Kunz, Griese, and Busse (2003) have suggested that the main etiology of unilateral paramedian pontine infarction is BAD. Nakase, Yamamoto, Ooiwa, Hayashi, and Nakajima (2000) also show that DM is associated with pontine infarction due to branch atherosclerosis, but is unrelated to the BSIs of lipid degeneration. These studies are consistent with our current findings.

Our study also showed that the poor prognosis of PPD was closely related to HbA1c, in contrast to the effect of other traditional vascular risk factors. Although the definite mechanism is unknown, pathogenetic condition and outcome of acute BAD was independently associated with HbA1c, which may indicate BAD may be an important feature in patients with DM. At first, poorly controlled DM causes autonomic neuropathy which increased the vulnerability of vertebrobasilar arteries which have less sympathetic vascular innervation, accelerates atherogenesis (Beausang‐Linder & Bill, 1981), and makes impairment of upstream smaller vessels more serious; secondly, the possible mechanism of progressive dyskinesia and poor prognosis with basilar artery branch disease is thought to be the reduction in perfusion caused by atherosclerotic lesions in the perforation large‐diameter penetrating arteries or from diffuse thrombus caused by atherosclerotic lesions (Bassetti et al., 1996). The PPA are 200–300μ min diameter and have less variation (Aronson, 1973). In the presence of parental atherosclerotic thrombosis, the smaller and shorter perforating vessels may be more susceptible to occlusion (Kim et al., 2012; Peress, Kane, & Aronson, 1973). Future researches are expected to explain the exact mechanism of the correlation with PPD and HbA1c. It should be noted that our study also found that PPD was associated with high relative frequency of basilar artery stenosis than LD.

There are some limitations in this study: (i) The diagnosis of BAD was actually based on the definition of radiology; (ii) Bias is inevitable in retrospective; (iii) The distinction between BAD and lacunar infarcts is not clear at all times.

Nonetheless, our major findings were that HbA1c levels were associated with BAD of the PPA and the higher levels of plasma HbA1c were strongly associated with poor prognosis of BAD in PPA territory. These results suggested that elevated HbA1c may be a more helpful serologic marker in the evaluation of pathogenetic condition and prognosis of BAD in PPA territory. With chronic exposure to raised concentration of glucose, decreased blood flow or enlarged thrombosis caused by atherosclerotic lesions, may lead to poor prognosis of BAD in the proximal penetrating artery. These findings may help in the selection of glucose management and the improvement of diabetes to prevent the progression of movement disorder or lowering the adverse prognosis of PPD.

## CONFLICT OF INTERESTS

There are no competitive interests between the authors.

## References

[brb3946-bib-0001] Aronson, S. M. (1973). Intracranial vascular lesions in patients with diabetes mellitus. Journal of Neuropathology & Experimental Neurology, 32, 183–196. 4268157 10.1097/00005072-197304000-00001

[brb3946-bib-0002] Barr, R. , Nathan, D. , Meigs, J. , & Singer, D. (2002). Tests of glycemia for the diagnosis of type 2 diabetes mellitus. Annals of Internal Medicine, 137(4), 263–272. 12186517 10.7326/0003-4819-137-4-200208200-00011

[brb3946-bib-0003] Bassetti, C. , Bogousslavsky, J. , & Barth, A. (1996). Isolated infarcts of the pons. Neurology, 46(1), 165–175. 8559368 10.1212/wnl.46.1.165

[brb3946-bib-0004] Baumgartner, R. W. , Sidler, C. , Mosso, M. , & Georgiadis, D. (2003). Ischemic lacunar stroke in patients with and without potential mechanism other than small‐artery disease. Stroke, 34, 653–659. https://doi.org/10.1161/01.STR.0000058486.68044.3B 10.1161/01.STR.0000058486.68044.3B 12624287

[brb3946-bib-0005] Beausang‐Linder, M. , & Bill, A. (1981). Cerebral circulation in acute arterial hypertension protective effects of sympathetic nervous activity. Acta Physiologica, 111, 193–199. 10.1111/j.1748-1716.1981.tb06724.x 7282395

[brb3946-bib-0006] Caplan, L. R. (1989). Intracranial branch atheromatous disease: A neglected, understudied, and underused concept. Neurology, 39, 1246–1250. https://doi.org/10.1212/WNL.39.9.1246 2671793 10.1212/wnl.39.9.1246

[brb3946-bib-0007] Duncan, P. , Jorgensen, H. , & Wade, D. (2000). Outcome measures in acute stroke trials: A systematic review and some recommendations to improve practice. Stroke, 31(6), 1429–1438. 10835468 10.1161/01.str.31.6.1429

[brb3946-bib-0008] Egoldstein, D. (2004). Tests of glycemia in diabetes. Diabetes Care, 18, 1761–1773. https://doi.org/10.2337/diacare.27.7.1761 10.2337/diacare.27.7.1761 15220264

[brb3946-bib-0009] Fisher, C. M. (1979). Capsular infarcts: The underlying vascular lesions. Archives of Neurology, 36, 65–73. https://doi.org/10.1001/archneur.1979.00500380035003 420625 10.1001/archneur.1979.00500380035003

[brb3946-bib-0010] Heo, S. H. , Lee, S. , Kim, B. J. , Kang, B. S. , & Yoon, B. W. (2010). Does glycated hemoglobin have clinical significance in ischemic stroke patients? Clinical Neurology and Neurosurgery, 112, 98–102. https://doi.org/10.1016/j.clineuro.2009.08.024 19766387 10.1016/j.clineuro.2009.08.024

[brb3946-bib-0011] Ichikawa, H. , Kuriki, A. , Kinno, R. , Katoh, H. , Mukai, M. , & Kawamura, M. (2012). Occurrence and clinicotopographical correlates of brainstem infarction in patients with diabetes mellitus. Journal of Stroke and Cerebrovascular Diseases, 21, 890–897. https://doi.org/10.1016/j.jstrokecerebrovasdis.2011.05.017 21757374 10.1016/j.jstrokecerebrovasdis.2011.05.017

[brb3946-bib-0012] Ichikawa, H. , Mukai, M. , Hieda, S. , Kamiya, Y. , Akizawa, T. , & Kawamura, M. (2010). Involvement of the basilar artery in diabetes mellitus: An MRI study of brainstem infarctions. European Neurology, 64, 230–235. https://doi.org/10.1159/000319924 20814216 10.1159/000319924

[brb3946-bib-0013] Ichikawa, H. , Mukai, M. , Takahashi, N. , Katoh, H. , Kuriki, A. , & Kawamura, M. (2009). Dilative arterial remodeling of the brain with different effects on the anterior and posterior circulation: An MRI study. Journal of the Neurological Sciences, 287, 236–240. https://doi.org/10.1016/j.jns.2009.06.029 19695577 10.1016/j.jns.2009.06.029

[brb3946-bib-0014] Kaps, M. , Klostermann, W. , Wessel, K. , & Bruckmann, H. (1997). Basilar branch disease presenting with progressive pure motor stroke. Acta Neurologica Scandinavica, 96(5), 324–327. https://doi.org/10.1212/WNL.46.1.165 9405003 10.1111/j.1600-0404.1997.tb00291.x

[brb3946-bib-0015] Kim, J. S. , Nah, H. W. , Park, S. M. , Kim, S. K. , Cho, K. H. , Lee, J. , … Kim, E. G. (2012). Risk factors and stroke mechanisms in atherosclerotic stroke: Intracranial compared with extracranial and anterior compared with posterior circulation disease. Stroke, 43, 3313–3318. https://doi.org/10.1161/STROKEAHA.112.658500 23160885 10.1161/STROKEAHA.112.658500

[brb3946-bib-0016] Kloppenborg, R. P. , Nederkoorn, P. J. , Graaf, Y. V. D. , & Geerlings, M. I. (2011). Homocysteine and cerebral small vessel disease in patients with symptomatic atherosclerotic disease. The SMART‐MR study. Atherosclerosis, 216, 461–466. https://doi.org/10.1016/j.atherosclerosis.2011.02.027 21411090 10.1016/j.atherosclerosis.2011.02.027

[brb3946-bib-0017] Kunz, S. , Griese, H. , & Busse, O. (2003). Etiology and long‐term prognosis of unilateral paramedian pontine infarction with progressive symptoms. European Neurology, 50(3), 136–140. 14530618 10.1159/000073053

[brb3946-bib-0018] Men, X. , Li, J. , Zhang, B. , Zhang, L. , Li, H. , & Lu, Z. (2013). Homocysteine and C‐Reactive protein associated with progression and prognosis of intracranial branch atheromatous disease. PLoS One, 8, e73030 https://doi.org/10.1371/journal.pone.0073030 24039853 10.1371/journal.pone.0073030 PMC3770607

[brb3946-bib-0019] Men, X. , Wu, A. , Zhang, B. , Li, H. , Zhang, L. , Chen, S. , … Lu, Z. (2013). Leukoaraiosis and NIHSS score help to differentiate subtypes of intracranial branch atheromatous disease in Southern Han Chinese patients with stroke. Neurological Sciences, 34, 1727–1733. https://doi.org/10.1007/s10072-013-1322-z 23430170 10.1007/s10072-013-1322-z

[brb3946-bib-0020] Nakase, T. , Yamamoto, Y. , Ooiwa, K. , Hayashi, M. , & Nakajima, K. (2000). Diabetes mellitus and ischemic stroke. Ischemic stroke topography for diabetes mellitus. Nosotchu, 22, 335–342. https://doi.org/10.3995/jstroke.22.335

[brb3946-bib-0021] Peress, N. S. , Kane, W. C. , & Aronson, S. M. (1973). Central nervous system findings in a tenth decade autopsy population. Progress in Brain Research, 40, 473–483. https://doi.org/10.1016/S0079-6123(08)60707-4 4802990 10.1016/S0079-6123(08)60707-4

[brb3946-bib-0022] Roquer, J. , Rodríguezcampello, A. , Cuadradogodia, E. , Giraltsteinhauer, E. , Jiménezconde, J. , Soriano, C. , & Ois, A. (2014). The role of HbA1c determination in detecting unknown glucose disturbances in ischemic stroke. PLoS ONE, 9, e109960 https://doi.org/10.1371/journal.pone.0109960 25485761 10.1371/journal.pone.0109960 PMC4259295

[brb3946-bib-0023] van Swieten, J. , Koudstaal, P. J. , Visser, M. , Schouten, H. , & van Gijn, J. (1988). Interobserver agreement for the assessment of handicap in stroke patients. Stroke, 19(5), 604–607. 3363593 10.1161/01.str.19.5.604

[brb3946-bib-0024] Tatu, L. , Moulin, T. , Bogousslavsky, J. , & Duvernoy, H. (1998). Arterial territories of the human brain: Cerebral hemispheres. Neurology, 50, 1699–1708. https://doi.org/10.1212/WNL.50.6.1699 9633714 10.1212/wnl.50.6.1699

[brb3946-bib-0025] Terai, S. , Hori, T. S. , Tamaki, K. , & Saishoji, A. (2000). Mechanism in progressive lacunar infarction: A case report with magnetic resonance imaging. Archives of Neurology, 57, 255–258. 10681085 10.1001/archneur.57.2.255

[brb3946-bib-0026] Uluduz, D. , Ince, B. , & Bozluolcay, M. (2005). Stroke patterns, etiology, and prognosis in patients with diabetes mellitus. Neurology, 64, 1558–1562. 15699413

[brb3946-bib-0027] Yamamoto, Y. , Ohara, T. , Hamanaka, M. , Hosomi, A. , Tamura, A. , & Akiguchi, I. (2011). Characteristics of intracranial branch atheromatous disease and its association with progressive motor deficits. Journal of the Neurological Sciences, 304, 78–82. https://doi.org/10.1016/j.jns.2011.02.006 21402390 10.1016/j.jns.2011.02.006

